# A Five‐Year Retrospective Analysis of Neoadjuvant Therapy in Early‐Stage Breast Cancer: Subtype Outcomes and Imaging Concordance

**DOI:** 10.1155/tbj/9197063

**Published:** 2026-03-31

**Authors:** Sohail Rooman Javed, Karen DeSouza, Ubaid Tanzim, Diego Ottaviani, Rebecca Roylance, Fharat Raja, Thomas Walsh, Prodromos Koutoukoglou, Chiara Creed, Ewan McFarlane, Sanjana Mathur, Arun Mahapatra, Elisavet Papadimitraki

**Affiliations:** ^1^ University College London Hospital NHS, London, UK; ^2^ University College London, London, UK, ucl.ac.uk

## Abstract

Although neoadjuvant systemic treatment (NST) is well established in the management of early breast cancer, there are limited clinical data available beyond the highly selected population evaluated in clinical trials. In this study, we assessed real‐world outcomes of NST in early breast cancer through a retrospective review of 201 patients treated between November 2019 and February 2025. Pathological complete response (pCR) rates were highest in HER2‐positive (60.2%) and triple‐negative (53.1%) breast cancer subtypes and lowest in those who were identified as oestrogen receptor‐positive (ER‐positive)/HER2‐negative (11.1%). In HER2‐positive disease, pCRs were more frequent in ER‐negative (75.8%) than ER‐positive (50.0%) cases, while pCR rates in triple‐negative breast cancer were similar with and without immunotherapy. Baseline positron emission tomography/computed tomography (PET/CT) upstaged 37% (35/94) of patients and showed 87% concordance with pathology, whereas magnetic resonance imaging (MRI) demonstrated a false‐negative rate of 17% and a false‐positive rate of 1.1%. In these cases, MRI results suggested residual disease despite a complete pathological response, with discrepancies varying by tumour subtype. Forty‐two percent of the patients underwent chemotherapy dose reductions; 12% required hospitalisation. NST was effective across aggressive subtypes, with pCR rates comparable to those observed in clinical trials. In summary, our results highlighted the contributions of functional imaging with PET/CT and MRI to staging, treatment response assessments and surgical planning in real‐world clinical practice.

## 1. Introduction

Breast cancer is the most common malignancy among women worldwide and remains a leading cause of cancer‐related mortality [1]. Neoadjuvant systemic treatment (NST) has become an integral part of the multidisciplinary management of early and locally advanced breast cancer, offering several clinical benefits. These include downstaging tumours to facilitate breast‐conserving surgery, enabling an earlier assessment of therapeutic responses and tailoring escalation or de‐escalation of adjuvant therapy [2]. The pathological complete response (pCR) achieved with NST serves as a surrogate marker for improved survival, particularly in specific subtypes such as HER2‐positive/oestrogen‐receptor (ER)‐negative and triple‐negative breast cancer (TNBC) [3].

Several key trials have established the role of NST in specific breast cancer subtypes. The NeoSphere trial evaluated the efficacy of neoadjuvant pertuzumab and trastuzumab combined with docetaxel in HER2‐positive breast cancer [4]. The trial demonstrated a pCR rate of 46% in patients receiving this combination, highlighting the value of dual HER2 blockade in this setting. Importantly, NeoSphere also emphasised the prognostic significance of achieving pCR, which was associated with improved progression‐free survival. Similarly, the TRYPHAENA study provided insights into the efficacy of dual HER2 inhibition with pertuzumab and trastuzumab when administered with anthracycline‐ or carboplatin‐based chemotherapy, reporting pCR rates of up to 66% with favourable tolerability [5].

For TNBC, the KEYNOTE‐522 trial established the benefit of combining immunotherapy with neoadjuvant chemotherapy (NACT). This study evaluated pembrolizumab plus chemotherapy and demonstrated significantly higher pCR rates (63.4% vs. 56.2% with chemotherapy alone, absolute difference ∼7%) and improved event‐free survival and overall survival [6–8]. These findings supported a new standard of care in TNBC patients [9, 10].

Despite the compelling results of these pivotal trials, real‐world evidence is limited, particularly regarding the use of NST in specific breast cancer subtypes, the integration of imaging modalities to predict response and the tolerability of these regimens outside controlled clinical trial settings.

Magnetic resonance imaging (MRI) data have been shown to correlate well with pathological responses, with a sensitivity and specificity of up to 88% and 69%, respectively, while positron emission tomography/computed tomography (PET/CT) is increasingly utilised for staging and treatment planning [11, 12]. However, the precise contributions of these imaging modalities to routine clinical practice require further evaluation. Recent national and international guidelines increasingly support the use of ^18^F‐fluorodeoxyglucose PET (FDG‐PET) to assess both initial staging and treatment responses in breast cancer [13]. Compared to conventional imaging, FDG‐PET offers enhanced accuracy and detail in evaluating the extent of disease and therapeutic response, contributing to more informed and individualised patient management [14]. The results of one recent study [15] revealed that staging PET/CT identified oligometastatic disease in 10% of cases of locally advanced disease.

In our centre, FDG‐PET/CT is incorporated into the neoadjuvant pathway in selected patients following multidisciplinary discussion, particularly when conventional imaging yields indeterminate findings, in cases of locally advanced or inflammatory disease, or in higher‐risk biological subtypes such as TNBC and HER2‐positive breast cancer. In this context, PET/CT may provide additional metabolic information that can be used to refine staging, guide biopsy decisions and support treatment planning before and after neoadjuvant therapy. However, the contributions of PET/CT to routine response assessment and surgical or radiotherapy planning remain incompletely defined, and real‐world data describing its clinical utility in contemporary neoadjuvant pathways remain limited.

This study describes the real‐world experience using NST at a single cancer centre, University College London Hospital (UCLH), across all breast cancer phenotypes, including HER2‐positive, ER‐positive and TNBC. The objectives include evaluating pCR rates with different chemotherapy regimens, the prognostic impact of imaging findings (MRI and PET/CT) and the toxicity profiles of various neoadjuvant regimens. By comparing our findings with results from pivotal trials, we aim to evaluate our centre’s current neoadjuvant treatment pathway, including regimen selection and response assessments compared with international standards.

## 2. Materials and Methods

We conducted a retrospective review of electronic medical records for patients with early breast cancer who received NST followed by breast surgery at UCLH between November 2019 and February 2025. Electronic medical records were obtained through EPIC, the electronic patient record system used at UCLH. Patients who received NST (all or in part) at another hospital were excluded from the analysis. Missing data were limited to selected covariates. Analyses involving variables with missing covariate data were performed using available‐case analysis without imputation, and the extent of missingness is reported in the relevant tables. The study size was determined by the number of consecutive eligible patients treated within the study period.

This is a retrospective analysis and review of routinely collected clinical data. Clinical data were retrieved from the local electronic patient database and anonymised for further analysis with the purpose of standard‐of‐care service evaluation. According to the National Institute for Health and Care Research in the United Kingdom (UK), retrospective data analysis for service evaluation is exempt from ethics committee approval. As this was a retrospective evaluation of real‐world clinical practice, no formal sample size calculation was performed.

The following data were collected from the patients’ medical records and organised into the following categories:

1. Patient demographics: Age, gender, screening or self‐detected tumour, body mass index (BMI) and menopausal status, defined as age over 60 or, for younger women, self‐reported cessation of menses for > 12 months prior to NST in the absence of bilateral oophorectomy or gonadal radiation therapy, hormone replacement therapy, endocrine therapies, or other factors that may affect menstrual status.
2. Clinical stage and tumour grade: Data on clinical tumour, nodes, metastasis (TNM) status at the time of initial diagnosis before any surgical intervention or systemic therapy, determined by physical examination, radiological studies and biopsy findings, were collected. Clinical staging was defined by the American Joint Committee on Cancer Staging Manual (8th edition).
 ER and progesterone receptor status were evaluated and reported using the Quick Allred score system, as documented in the histology report from the initial core biopsy of breast and/or axillary tissue. ER‐negativity was defined as an ER score of 0–2, and ER‐positivity, as ≥ 3.
 HER2 status was assessed using immunohistochemical analysis (IHC) and confirmed by fluorescence in situ hybridisation (FISH) for cases with an equivocal IHC score of 2+.
 TNBC was defined as ER‐negative (Quick score ≤2) and HER2‐negative disease (IHC score of 0 or 1+, or 2+ without amplification on FISH).
 Tumour histology, grade and Ki‐67/Mib‐1 scores were also collected.
3. Surgical procedures and pathological outcomes: Details on the type of breast and axillary surgery, pathological stage at surgery and pathological outcomes were recorded. A pCR was defined as the absence of invasive cancer in both the breast and axilla as documented in the final pathology report. Patients with residual ductal carcinoma in situ (DCIS) (ypTis) were scored as having achieved pCR.
4. Imaging and radiological assessments: Results from pretreatment MRIs were compared with post‐NST MRIs, including change in tumour volume and evidence of radiological complete response. Radiological complete response was defined as documentation of no residual enhancing disease at the primary tumour site in the final preoperative MRI report.
 The reports of the scans were reviewed and compared with the final surgical pathological results, and any discordance in radiological and pathological findings was assessed and categorised. If the extent of post‐NST disease was underestimated by imaging (i.e., MRI showed no disease, but pathology identified residual disease), the result was categorised as false negative for residual disease. In cases in which the extent of post‐treatment disease was overestimated by imaging (i.e., MRI suggested residual disease, but pathology showed pCR), the findings were categorised as false positive for residual disease.
 Data were collected from PET/CT reports, focusing on: (1) detection of regional nodal involvement (internal mammary, supraclavicular and subpectoral nodes); (2) identification of distant metastatic disease, including cases that fell outside standard PET/CT indications (e.g., patients without stage III disease or inflammatory breast cancer but with indeterminate CT findings) and (3) metabolic response assessment on pre‐surgical PET scans. If conventional CT imaging was available, the findings would be compared with PET/CT results to identify differences in the accuracy of nodal and metastatic detection, as these differences could have an impact on disease staging and treatment planning. Additionally, presurgical PET results were gathered, and rates of partial metabolic response or complete metabolic response (CMR) were compared against final surgical pathology outcomes to evaluate the accuracy of PET imaging for predicting pathological responses. Formal standardised metabolic response criteria (e.g., PERCIST) were not systematically applied. PET response classifications were largely based on narrative clinical report interpretations.
5. NST regimen: Data were collected on the selection of NST regimens, chemotherapy dose reductions, dose interruptions and hospitalisations. Additionally, for patients who received neoadjuvant immunotherapy, information was gathered on immune‐related adverse events (irAEs), including their incidence, management with steroids and hospitalisation rates. Chemotherapy and immunotherapy‐related dose reductions, treatment interruptions and hospitalisations were documented to evaluate tolerability and treatment adherence.

### 2.1. Outcomes and Endpoints

The primary endpoint is pCR, defined as the absence of invasive cancer in both the breast and axilla (*ypT0/is ypN0*). This definition was applied uniformly across all breast cancer subtypes and throughout the study period. Patients with residual DCIS (*ypTis*) were still considered to have achieved pCR. The pCR rates were compared across the three major breast cancer subtypes: ER‐positive, HER2‐negative, HER2‐positive and TNBC.

Exploratory analyses were conducted within key biological subgroups. Among HER2‐positive breast cancer cases, the influence of ER expression on pCR rates was examined. In the TNBC cohort, pCR rates were compared between patients who did and did not receive immunotherapy. A sensitivity analysis restricted to KEYNOTE‐522‐eligible patients (≥ T2 or node‐positive disease) was performed to reduce stage‐related confounding issues in comparisons of immunotherapy‐treated and untreated TNBC patients.

### 2.2. Secondary Endpoints

a. Safety and tolerability: As assessed by dose reductions, treatment discontinuation and hospitalisation rates. An exploratory evaluation of safety outcomes was also undertaken in TNBC patients who received immunotherapy.
b. Imaging accuracy: Concordance between imaging modalities (MRI and FDG‐PET/CT) and final pathological outcomes was assessed. Concordance rates were also examined across the three breast cancer subtypes. In addition, an exploratory evaluation of FDG‐PET/CT utility in detecting extra‐axillary nodal involvement as a marker of occult metastatic disease was undertaken.

### 2.3. Statistical Analysis

Data analysis was performed using Python (version 3.8), employing the following libraries: pandas 1.3.0, numpy 1.20.0, scipy 1.7.0, openpyxl 3.0.0 and statsmodels 0.12.0. Descriptive statistics were used to summarise patient demographics, clinical characteristics and treatment outcomes. Categorical variables were compared using the Chi‐square test. A *p*‐value of < 0.05 was considered statistically significant. For binary proportions such as pCR rates, 95% confidence intervals (CIs) were calculated using the Wilson score method to provide more accurate interval estimation, particularly in subgroups with small sample sizes.

## 3. Results

### 3.1. Patient Demographics

Two hundred one female patients were identified as having received NST followed by curative surgery at UCLH between November 2019 and February 2025. Median age at diagnosis was 50.79 years; 111 patients (55%) were premenopausal at the time of diagnosis and 90 (45%) were postmenopausal. Most of the patients (76%) were between 41 and 70 years of age. Grade 3 tumours were most common (65.7%). Twenty‐two patients were treated with NST in a clinical trial. Table 1 summarises demographic and clinical characteristics of each case by breast cancer subtype.

**TABLE 1:** Demographic and clinical characteristics of patients by breast cancer subtype.

	Total (*N* = 201) (%)	HER2‐positive (*n* = 83) (%)	TNBC (*n* = 64) (%)	ER‐positive HER2‐negative (*n* = 54) (%)
Number of patients	201	83 (41.3)	64 (31.8)	54 (26.9)

*Age*				
> 70	15 (7.5)	7 (8.4)	5 (7.8)	3 (5.6)
41–70	152 (75.6)	61 (73.5)	49 (76.6)	42 (77.8)
≤ 40	34 (16.9)	15 (18.1)	10 (15.6)	9 (16.7)

*Tumour size*				
T1	14 (7.0)	4 (4.8)	8 (12.5)	2 (3.7)
T2	115 (57.2)	52 (62.7)	39 (60.9)	24 (44.4)
T3	48 (23.9)	22 (26.5)	11 (17.2)	15 (27.8)
T4	24 (11.9)	5 (6.0)	6 (9.4)	13 (24.1)

*Nodal status*				
N0	76 (37.8)	30 (36.1)	32 (50.0)	14 (25.9)
N1	83 (41.3)	31 (37.3)	26 (40.6)	26 (48.1)
N2	15 (7.5)	7 (8.4)	1 (1.6)	7 (13.0)
N3	27 (13.4)	15 (18.1)	5 (7.8)	7 (13.0)

*Tumour histology*				
IDC	193 (96.0)	78 (94.0)	62 (96.9)	53 (98.1)
ILC	7 (3.5)	5 (6.0)	1 (1.6)	1 (1.9)
Other	1 (0.5)	0 (0.0)	1 (1.6)	0 (0.0)

*Tumour grade*				
G1	2 (1.0)	0 (0.0)	0 (0.0)	2 (3.7)
G2	65 (32.3)	29 (34.9)	8 (12.5)	28 (51.9)
G3	132 (65.7)	54 (65.1)	54 (84.4)	24 (44.4)
Unknown	2 (1.0)	0 (0.0)	2 (3.1)	0 (0.0)

*Ki-67*				
Low (≤ 5)	12 (6.0)	3 (3.6)	2 (3.1)	7 (13.0)
Intermediate (6–29)	54 (26.9)	30 (36.1)	9 (14.1)	15 (27.8)
High (> 30)	88 (43.8)	32 (38.6)	40 (62.5)	16 (29.6)
Not documented	47 (23.4)	18 (21.7)	13 (20.3)	16 (29.6)

*Menopausal status*				
Premenopausal	111 (55.2)	46 (55.4)	34 (53.1)	31 (57.4)
Menopausal	90 (44.8)	37 (44.6)	30 (46.9)	23 (42.6)

*Smoker*				
Current	32 (15.9)	8 (9.6)	10 (15.6)	14 (25.9)
Ex‐smoker	37 (18.4)	22 (26.5)	8 (12.5)	7 (13.0)
Nonsmoker	132 (65.7)	53 (63.9)	46 (71.9)	33 (61.1)

*BMI*				
< 25	88 (43.8)	40 (48.2)	30 (46.9)	18 (33.3)
25 ≤; < 30	62 (30.8)	27 (32.5)	17 (26.6)	18 (33.3)
≥ 30	51 (25.4)	16 (19.3)	17 (26.6)	18 (33.3)

*Presentation*				
Screening	27 (13.4)	11 (13.3)	11 (17.2)	5 (9.3)
Symptomatic	174 (86.6)	72 (86.7)	53 (82.8)	49 (90.7)

*Surgery*				
WLE + SNB	48 (23.9)	20 (24.1)	21 (32.8)	7 (13.0)
WLE + ANC	31 (15.4)	14 (16.9)	10 (15.6)	7 (13.0)
Mx + SNB	42 (20.9)	18 (21.7)	17 (26.6)	7 (13.0)
Mx + ANC	80 (39.8)	31 (37.3)	16 (25.0)	33 (61.1)

Abbreviations: ANC = axillary node clearance, Mx = mastectomy, SNB = sentinel node biopsy, WLE = wide local excision.

Seventy‐six (38%) patients were node negative at initial diagnosis, and 193 (96%) presented with an invasive ductal carcinoma histological subtype. Most patients (174 [87%]) initially presented with symptomatic breast cancer rather than having been identified by screening.

### 3.2. Breast Cancer Subtypes

The breakdown by cancer subtype was as follows: 83 (41.3%) with HER2‐positive disease, 64 (31.8%) with TNBC and 54 (26.9%) with ER‐positive/HER2‐negative disease. HER2‐positive disease was confirmed in 70 patients with an IHC score of 3+ and in 12 patients with an IHC score of 2+ and FISH‐positivity. Amongst the 83 patients with HER2‐positive disease, 34 (41%) had ER‐negative/HER2‐positive disease and 49 (59%) had ER‐positive/HER2‐positive disease.

ER‐positive tumours had a higher proportion of T4 disease (24.1%) compared to HER2‐positive and triple‐negative subtypes. This finding is consistent with clinical guidelines, where neoadjuvant therapy is often reserved for larger ER‐positive tumours that require downstaging prior to surgery [10, 16]. TNBC cases featured a significantly higher proportion of node‐negative (N0) disease at baseline (50%) compared to other subtypes. Compared to ER‐positive and HER2‐positive groups, TNBC cases also demonstrated a higher frequency of Grade 3 tumours (84.4%) and high proliferative index, with 59.4% showing Ki‐67 levels greater than 30%.

Amongst HER2‐positive patients, 82/83 received dual anti‐HER2 therapy with trastuzumab and pertuzumab (administered intravenously or subcutaneously). This was combined with various chemotherapy regimens containing anthracycline, taxanes, platinum and cyclophosphamide. The most common backbone was an anthracycline–taxane sequence: 57/83 (68.7%) received epirubicin 90 mg/m^2^ and cyclophosphamide 600 mg/m^2^ every 21 days for three cycles, followed by 12 weekly doses of paclitaxel 80 mg/m^2^ (with eight total planned cycles). Other regimens used earlier included an anthracycline‐based sequence of fluorouracil 500 mg/m^2^, epirubicin 100 mg/m^2^ and cyclophosphamide 500 mg/m^2^ (FEC) every 21 days for three cycles, followed by docetaxel 75 mg/m^2^ every 21 days for three cycles with trastuzumab and pertuzumab. In 8% of patients, weekly paclitaxel (80 mg/m^2^) was used as an alternative to docetaxel. A nonanthracycline regimen was used in patients with previous exposure to anthracyclines or contraindication to anthracyclines. This regimen was given to 17% of patients, consisting of docetaxel 75 mg/m^2^ plus carboplatin (area‐under‐curve 6) every 21 days for six cycles with trastuzumab and pertuzumab.

Amongst the TNBC patients, 27 (42%) received immunotherapy as per the KEYNOTE‐522 regimen. When NST included chemotherapy only, the most common regimen was paclitaxel–carboplatin, followed by FEC‐T (fluorouracil, epirubicin, cyclophosphamide–docetaxel) or EC (epirubicin–cyclophosphamide).

For the ER‐positive HER2‐negative patients, the most common regimen was EC‐paclitaxel (83%), followed by FEC‐T and FEC‐docetaxel/cyclophosphamide (TC). Across all subtypes, 5‐fluorouracil (5FU) was dropped from standard regimens during the data collection period, reflecting a change in institutional practice in line with emerging data showing limited additional benefit from 5FU in modern anthracycline‐ and taxane‐based NACT regimens [17].

A comparison of clinical and pathological characteristics of tumours, as well as demographics according to breast cancer subtype, is shown in Table 1.

### 3.3. Primary Endpoint: Pathological Response Rates

In the full dataset, 44.8% of cases achieved pCR (95% CI 38.1%–51.7%), with pCR rates varying across subtype from 11.1% (95% CI 5.2%–22.2%) in ER‐positive/HER2‐negative breast cancer, 53.1% (95% CI 41.1%–64.8%) in TNBC, and 60.2% (95% CI 49.5%–70.1%) in HER2‐positive breast cancer (Figure 1(a)).

FIGURE 1pCR rates across (a) BC subtypes and (b) ER scores.

**Figure:**
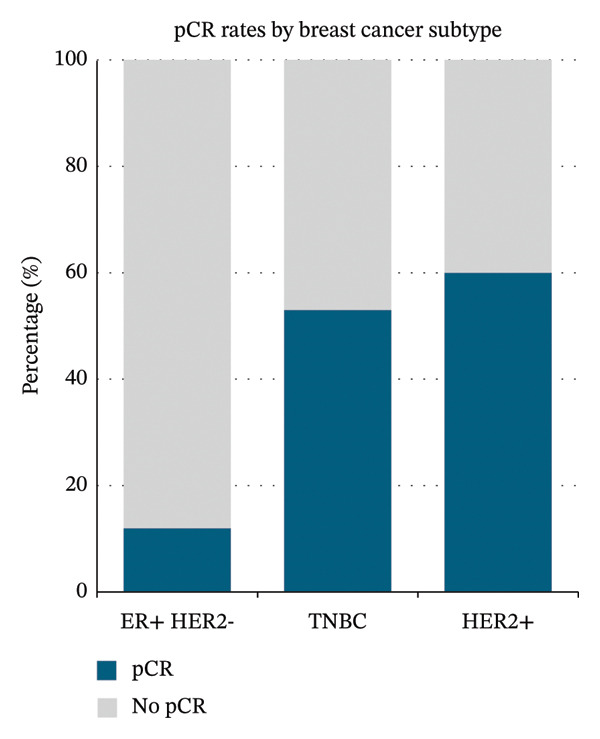
(a)

**Figure:**
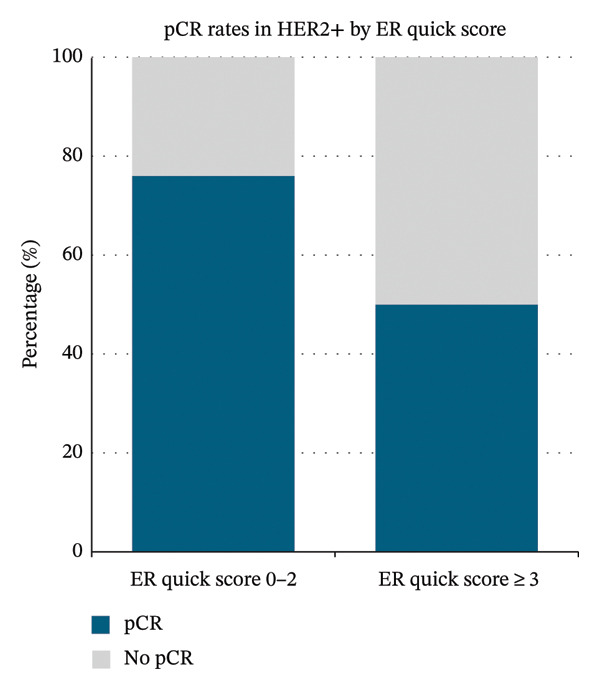
(b)

### 3.4. Exploratory Analysis: pCR Rates in HER2‐Positive Patients Stratified by ER Allred Score

Among HER2‐positive patients, pCR rates appeared to vary according to ER expression. Patients with low or absent ER expression (ER score 0–2) demonstrated higher pCR rates than those with higher ER expression (ER score > 3) (75.8% vs. 50.0%; Figure 1(b)). ER‐negative status (ER score 0–2) was associated with significantly greater odds of achieving pCR than ER‐positive disease (ER score 3–8), with an odds ratio of 3.13 (95% CI, 1.18–8.30; *p* = 0.01).

A detailed breakdown shows a trend of decreasing pCR rates with increasing ER expression (Table 2).

**TABLE 2:** pCR rates by ER status and score in HER2‐positive patients.

ER status	Number of patients	pCR (*n*)	pCR rate (%)	95% CI
ER‐negative (score 0–2)	33	25	75.8	59.0–87.2
ER‐positive (score 3–8)	48	24	50.0	36.4–63.6
*p*‐value	0.01			

### 3.5. Exploratory Analysis: TNBC With and Without Immunotherapy

The pCR rates between TNBC patients who did (*n* = 27) and did not (*n* = 37) receive immunotherapy were comparable (51.9% vs. 54.1%), with an odds ratio of 0.92 (95% CI, 0.34–2.47; *p* ≥ 0.05) (Table 3).

**TABLE 3:** pCR rates in TNBC with and without immunotherapy.

	Number of patients	pCR (*n*)	pCR rate %	95% CI	*p*‐value
TNBC with immunotherapy	27	14	51.9	34.0–69.3	1.00
TNBC without immunotherapy	37	20	54.1	38.4–69.0	—
TNBC without immunotherapy (KEYNOTE‐eligible only)	29	13	45.0	27.3–63.7	—
All TNBC	64	34	53.1	41.1–64.8	—

A comparison of demographic characteristics between TNBC patients treated with or without pembrolizumab showed generally similar profiles (Table 4). Notably, no patients with T1N0 disease received neoadjuvant immunotherapy, which aligns with the nationally approved indication based on the KEYNOTE‐522 trial that restricts pembrolizumab use to tumours of at least T2 size or N positive if T1. When patients with T1 disease (*n* = 8) were excluded, the pCR rate was 45% (13/29) in those who did not receive immunotherapy compared to 51.9% (14/27) in those who did. The median year of treatment was later in the pembrolizumab cohort, reflecting evolving clinical practice and adoption of immunotherapy following KEYNOTE‐522.

**TABLE 4:** Baseline clinicopathological characteristics of TNBC patients treated with and without pembrolizumab.

	TNBC with immunotherapy	TNBC without immunotherapy	*p* value
Age at diagnosis (median, interquartile range [IQR])	55.6 (45.6–61.5)	50.6 (42.1–61.0)	0.399
BMI (median, IQR)	24.3 (21.9–29.2)	27.6 (23.3–30.9)	0.308
%Ki‐67‐positive (median, IQR)	54.0 (26.2–73.8)	50 (40.0–75.0)	0.712
Tumour size			0.06
T1	0	8	
T2	20	19	
T3	5	6	
T4	2	4	
Nodal status			0.856
N0	14	18	
N1	11	15	
N2	0	1	
N3	2	3	
Tumour grade			0.939
G2	3	5	
G3	24	32	
Year of treatment (median, IQR)	2022 (2021–2023)	2020 (2019–2022)	< 0.001

A sensitivity analysis was performed that excluded patients with T1 disease (*n* = 8) to reduce stage‐related confounding (Table 3). In this restricted cohort, the pCR rate was 45% (13/29) in patients who did not receive immunotherapy compared with 51.9% (14/27) in those who did. However, this comparison remained limited by a small sample size and should be interpreted cautiously.

### 3.6. Secondary Endpoint: Toxicities

Eighty‐five of 201 patients (42.3%) required at least one chemotherapy dose reduction of their chemotherapy during NST. The most common reasons were grade ≥ 2 neuropathy (38.1%), fatigue (26.2%), diarrhoea (11.9%) and cytopenias (10.7%). Thirty‐four of 201 patients (16.9%) did not complete the total initial intended number of cycles of neoadjuvant treatment: the reasons for this included therapy toxicity and patient choice. Twenty‐four of 201 (11.9%) patients were admitted to the hospital during neoadjuvant therapy. Table 5 describes toxicities according to breast cancer subtype.

**TABLE 5:** Treatment exposure and tolerability during neoadjuvant therapy by breast cancer subtype.

	Completion of planned neoadjuvant chemotherapy (%)	Dose reductions (%)	Dose delays (%)	Hospitalisation (%)
HER2‐positive	70/83 (84.3)	31/83 (37.3)	25/83 (30.1)	2/83 (2.4)
ER‐positive HER2‐negative	46/54 (85.2)	19/54 (35.2)	10/54 (18.5)	4/54 (7.4)
TNBC	51/64 (79.7)	33/64 (51.6)	30/64 (46.9)	18/64 (28.1)
TNBC with IO	21/27 (77.8)	14/27 (51.9)	12/27 (44.4)	12/27 (44.4)
TNBC without IO	30/37 (81.1)	19/37 (51.4)	18/37 (48.6)	6/37 (16.2)

**Pembrolizumab exposure within TNBC cohort (*n* = 27)**				
**Number of pembrolizumab doses received**	**Number of patients (*n*)**			

4	2			
5	2			
6	4			
7	6			
8	13			

### 3.7. Exploratory Analysis: Toxicity in TNBC Patients With Immunotherapy

• Five of 27 (18.5%) patients were prescribed steroids for IO‐related toxicities, including hepatitis, nephritis and adrenal insufficiency.
• Seven of 27 (25.9%) patients experienced Grade 3 (G3) toxicities, with reasons including fatigue, infection, cytopenia, hepatitis and diarrhoea.
• Twelve of 27 (44.4%) patients required hospitalisation, compared to 6/37 (16%) amongst TNBC who did not receive immunotherapy.
• Eighteen of 27 (66.7%) patients experienced irAEs with the following breakdown: skin toxicities, 10/27 (37.0%); endocrine dysfunction, 9/27 (33.3%); hepatitis, 4/27 (14.8%); gastrointestinal toxicity, 2/27 (7.4%); haematologic toxicity, nephritis, 1/27 (3.7%).
• Fourteen of 27 (52%) required at least one dose of pembrolizumab in the neoadjuvant phase due to an immune‐mediated toxicity.

### 3.8. Secondary Endpoint: Radiological Assessment

#### 3.8.1. MRI Scans and Correlations With pCR

One hundred seventy‐five patients (87%) underwent pre‐ and post‐NST MRI scans. The reports of the scans were reviewed and compared with the final surgical pathological results; false negatives (i.e., MRI showed complete radiological response while pathology revealed residual cancer) and false positives (MRI showed residual disease while pathology revealed pCR) were assessed. Discordance was assessed both in the breast and axilla.

**FIGURE 2:**
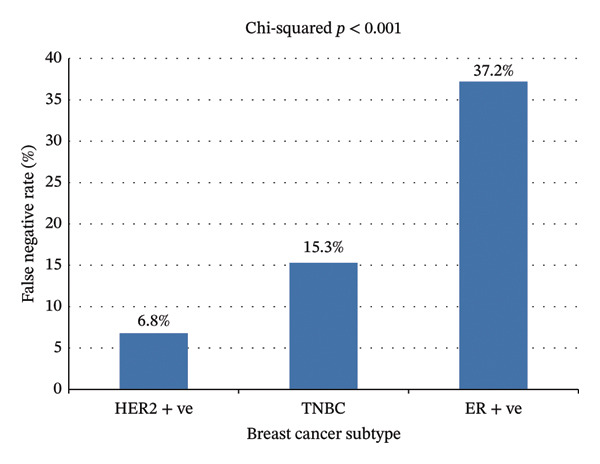
False‐negative rates of MRI according to BC subtypes.

The false‐negative rate was 21/175 (12%) in the axilla and 15/175 (8.5%) in the breast. The overall false‐negative rate was 28/175 (16%). The rates of MRI inaccuracy varied according to subtype, as shown in Figure 2(a). The overall false‐positive rate was 2/175; in both cases, imaging suggested either stable or progressive disease, while final pathology revealed a partial or complete pathological response.

### 3.9. Concordance of PET/CT Responses With Final Pathology

FDG‐PET/CT scans were performed in 94 patients (47%) in our cohort as part of initial staging. Most of these studies were performed during the last few years, reflecting a recent change in our practice. Tumour status and nodal size were significantly different between patients who did and did not undergo PET (Table 6).

**TABLE 6:** Patients undergoing baseline PET/CT vs no PET/CT.

	PET	No PET	*p*‐value
Age at diagnosis (median, IQR)	50.2 (42.0–57.7)	52.0 (43.4–59.9)	0.34
BMI (median, IQR)	26.4 (23.0–30.1)	25.6 (22.6–29.8)	0.54
Ki‐67% (median, IQR)	40 (18–60)	30 (15–60)	
Tumour size			0.001
T1	2	12	
T2	49	66	
T3	22	26	
T4	21	3	
Nodal status			0.001
N0	24	52	
N1	37	46	
N2	6	9	
N3	27	0	
Grade			0.22
G1	1	1	
G2	40	25	
G3	52	80	
Subtype			0.47
ER‐positive/HER2‐negative	28	26	
HER2‐positive	34	49	
TNBC	32	32	
Year of Treatment (median, IQR)	2022 (2021–2023)	2020 (2019–2022)	0.001

Twenty‐three patients underwent PET/CT scanning prior to surgery after completing NST. The results were compared with the initial staging PET scans to evaluate metabolic response in both the breast and lymph nodes. All reports indicated either a partial or a CMR in these two areas.

The results of PET/CT scans showed good correlation with final pathology findings in the breast, with 20 out of 23 cases (87%) demonstrating concordant results. In the remaining three cases, the PET scan indicated a CMR in the breast, but residual disease was found on final pathology.

In the lymph nodes, correlation was observed in 15 out of 23 cases (65%). In the other eight cases, the results of PET scans suggested a CMR in the lymph nodes, yet residual nodal disease was present on final pathology. Overall, when looking at the response in both the breast and nodal areas, 13/23 (56%) had concordant radiological and pathological responses. No significant differences were observed in different breast cancer subtypes, but the overall numbers were small. Discordance was observed between PET/CT and pathology in 4/32 (12.5%) cases of TNBC, in 2/35 (5.71%) cases of HER2‐positive BC and in 4/28 (14.29%) cases of ER‐positive/HER2‐negative BC.

### 3.10. Exploratory Analysis: Detection of Extra‐Axillary Nodal Involvement by FDG‐PET/CT

Reports were reviewed by our multidisciplinary team to determine whether PET/CT identified FDG‐avid internal mammary, subpectoral and/or supraclavicular fossa (SCF) nodes. Among these, 36 patients had FDG‐avid nodal involvement in one or more of these regions. Of these 36 patients, 24 had undergone conventional CT scanning prior to PET/CT. Notably, in nine of these 24 patients (37%), the nodal involvement was not detected on conventional imaging and was only identified on PET/CT, resulting in disease upstaging. In these cases, the PET/CT findings were reviewed prior to surgery and used to inform radiotherapy decisions, with five patients having their RT fields expanded to include internal mammary and/or SCF regions. Review of multidisciplinary team records suggests that PET/CT findings were considered during radiotherapy planning and likely contributed to decisions to expand fields to include internal mammary nodes and/or SCF regions in five patients. While it is possible that surgical findings also influenced these decisions, the sequence of events suggests that PET/CT played an important role rather than being the sole determinant of these decisions.

### 3.11. Exploratory Analysis: Detection of Metastatic Disease

Metastatic disease was identified in eight patients (8.5%), including seven on initial staging PET and one on post‐neoadjuvant PET. Six of these cases showed no metastasis on staging CT, while the other two had indeterminate CT findings, which were later confirmed as FDG‐avid on PET. Metastatic sites included bone (ribs, pelvis and spine) and visceral organs (liver, lungs and brain). In cases with rib involvement, radiotherapy fields were adjusted to include the affected area. Among the eight patients with metastatic disease, BC subtypes were roughly equally distributed across ER‐positive/HER2‐negative, HER2‐positive and triple‐negative disease. Given the small sample size, no meaningful correlation with subtype could be established.

## 4. Discussion

This retrospective analysis provides real‐world insight into the outcomes of the NST treatment pathway in early breast cancer at a single tertiary care centre over a 5‐year period. Changes in the treatment landscape over this period are reflected in the different treatment regimens. Our findings reaffirm the efficacy of NST in achieving pCR in an unselected population, particularly in patients with HER2‐positive and TNBC. Furthermore, our well‐established neoadjuvant treatment pathway with multidisciplinary input provides real‐world evidence for the evolving role of PET/CT imaging in informing treatment decisions for selected patients, such as those with indeterminate nodal disease.

### 4.1. Primary Outcome: pCR Rates and Subtype Comparisons

The overall pCR rate in this cohort was 44.8%, with the highest rates observed in HER2‐positive (60.2%) and TNBC (53.1%). These rates are largely consistent with published clinical trial data, such as NeoSphere and TRYPHAENA, which demonstrated pCR rates between 46% and 66% in patients with HER2‐positive breast cancer who were receiving dual anti‐HER2 blockade. In our cohort, HER2‐positive/ER‐negative patients achieved particularly high pCR rates (75.8%), supporting findings that ER‐negativity is a key predictor of response within this subgroup.

By contrast, although pCR rates in TNBC were relatively high (53.1%), they were lower than those reported in the KEYNOTE‐522 trial, where the addition of pembrolizumab to chemotherapy yielded pCR rates of 63.4% compared to 56.2% with chemotherapy alone. In the KEYNOTE‐522 trial, most patients (75%) had stage II disease. In our cohort of patients with stage II–III disease, we observed similar pCR rates in patients who received immunotherapy (51.9%) and those who did not (54.1%); however, this analysis was limited by a small sample size and should be interpreted with caution. In addition, patients receiving pembrolizumab were treated in more recent years, reflecting evolving clinical practice. These findings are therefore exploratory and contrast with the clear benefit of immunotherapy demonstrated in KEYNOTE‐522.

Interestingly, when T1 disease was excluded, the pCR rate was 46% amongst those who did not receive immunotherapy compared to 51.9% in those who did. It is noteworthy that grade 3 toxicity rates were higher in our cohort (25% vs. 13% in KEYNOTE‐522). This discrepancy between our findings and those of KEYNOTE‐522 may reflect real‐world differences in patient selection, discordance in receptor status assessments, differences in treatment adherence and sample size limitations. In addition, the higher immunotherapy‐associated toxicity experienced by our patients could have contributed to lower pCR rates through treatment interruptions or loss of dose density, although our current analysis did not specifically quantify this effect.

Recent evidence also highlights that, beyond the binary achievement of pCR, the pattern and burden of residual disease following neoadjuvant therapy carry important prognostic and therapeutic implications [18]. Patients with residual invasive disease represent a biologically heterogeneous group, with outcomes influenced by both tumour subtype and the distribution of residual tumour within the breast and axilla [18].

### 4.2. Secondary Outcome: Toxicity and Treatment Tolerability

The overall tolerability of NST regimens in this real‐world cohort was acceptable, although nearly 42% required at least one chemotherapy dose reduction, and 17% did not complete the intended course. These findings were more frequent than what was observed in clinical trials, particularly those including platinum agents and immunotherapy [19]. Amongst TNBC patients receiving pembrolizumab, irAEs were common, with 67% experiencing toxicity, 25% experiencing ≥ G3 toxicity and 44% requiring hospitalisation during their neoadjuvant treatment. These findings underscore the importance of multidisciplinary management and vigilance in toxicity monitoring during neoadjuvant immunotherapy.

### 4.3. Secondary Outcome: Role of Imaging in Response Assessment and Surgical Planning

Our results emphasise the evolving role of imaging in both staging and treatment planning. MRI was widely used pre‐ and post‐NST, with an overall false‐negative rate of 17%, indicating moderate concordance with pathological outcomes. This finding is consistent with prior literature that supports MRI as a useful, although not infallible, tool in assessing treatment responses [8]. Subtype‐based variability in MRI accuracy also underscores the need for refined interpretation frameworks. For example, MRI was less accurate in detecting residual disease in ER‐positive/HER2‐negative tumours, which often exhibit low cellularity and less conspicuous imaging features, compared to triple‐negative or HER2‐positive subtypes, where more pronounced radiological changes are seen. Our findings align with previous studies indicating that MRI frequently overestimates pathological responses, particularly in ER‐positive/Luminal A subtypes of breast cancer [20–22]. This suggests that interpretation of MRI response may need to be tailored by subtype, potentially incorporating additional imaging and/or biomarker data to improve accuracy.

Importantly, PET/CT identified extra‐axillary nodal involvement not seen on conventional imaging in 37% of cases, leading to upstaging and changes in radiotherapy fields for some patients. However, this finding should be interpreted with caution, as PET/CT was not uniformly performed across the cohort. Scans were typically requested at the clinician’s discretion, more often in patients with extensive nodal disease or cases in which MRI findings were equivocal. This introduces a potential selection bias, as patients undergoing PET/CT may have had a higher pretest probability of additional disease burden. Nonetheless, these findings highlight PET/CT’s utility as a sensitive staging modality, particularly in identifying occult nodal disease. Moreover, our findings suggest that results of end‐of‐treatment PET/CT may serve as potential markers of treatment response. Concordance with final pathology was seen in 87% of breast cases and 65% of nodal cases, suggesting a potential role for PET in predicting pCR and guiding surgical de‐escalation strategies or decisions about radiation therapy.

Given these findings, the results of PET/CT may contribute to multidisciplinary discussions focused on response assessments in selected patients. However, these observations should be considered hypothesis‐generating, and further prospective validation will be required before PET/CT can be used to inform surgical planning or radiotherapy decisions.

### 4.4. Staging and Imaging Implications for Surgical Decision‐Making

The improved imaging footprint afforded by MRI and PET/CT not only refines initial staging but may offer predictive insights into treatment response. The use of PET/CT holds promise as a biomarker of response, potentially aiding decisions around surgical de‐escalation, such as avoidance of axillary lymph node dissection in select patients. With increasing interest in tailoring surgery based on treatment response, particularly in HER2‐positive and TNBC subtypes, our findings support a more integrated imaging‐based approach to surgical planning.

### 4.5. Limitations

The strengths of this study include the breadth of subtype representation and detailed analysis of imaging concordance, toxicity and treatment adherence. However, limitations include its retrospective nature, single‐centre design and limited sample size in certain subgroups, particularly those receiving immunotherapy. Imaging assessments were derived from routine clinical reports and were not centrally reviewed, and formal standardised response criteria for MRI or PET/CT were not applied prospectively. PET/CT was performed in a selected population, with metabolic response interpretation based on narrative clinical reports rather than formal criteria such as PERCIST. In addition, axillary assessment in this study was based on MRI findings only. Although targeted axillary ultrasound may have been performed as part of routine care, ultrasound findings were not systematically collected or analysed.

## 5. Conclusions

This retrospective single‐centre study demonstrates that NST achieves pCR rates in routine clinical practice comparable to those reported in pivotal trials, particularly in HER2‐positive and TNBC. MRI showed moderate concordance with pathological outcomes, while FDG‐PET/CT contributed to staging and treatment planning in selected patients by identifying occult nodal and metastatic disease and demonstrating reasonable correlation with final pathology. Treatment‐related toxicities, particularly with immunotherapy‐containing regimens, were common and frequently required dose modification, highlighting the importance of careful monitoring and multidisciplinary management. Overall, these findings support the effectiveness of contemporary NST pathways in real‐world practice and demonstrate the evolving role of advanced imaging in guiding treatment and surgical decision‐making, while prospective validation in larger cohorts remains warranted.

## Author Contributions

Sohail Rooman Javed: conceptualisation, methodology, formal analysis, investigation, data curation, visualisation, and writing–original draft. Karen DeSouza: conceptualisation, methodology, investigation, data curation, and resources. Ubaid Tanzim: formal analysis. Diego Ottaviani: investigation and resources. Rebecca Roylance: investigation and resources. Fharat Raja: investigation and resources. Thomas Walsh: investigation. Prodromos Koutoukoglou: investigation. Chiara Creed: investigation. Ewan McFarlane: investigation and data curation. Sanjana Mathur: investigation and data curation. Arun Mahapatra: investigation and data curation. Elisavet Papadimitraki: conceptualisation, methodology, data curation, writing–original draft, writing–review and editing, supervision, funding acquisition, and resources.

## Funding

Funding was secured by the authors for a collaborative working project funded by Novartis UK Ltd.

## Disclosure

Funders played no role in the research. All authors read and approved the final manuscript.

## Ethics Statement

This is a retrospective analysis of routinely collected clinical data, conforming to a retrospective review. Clinical data were retrieved from the local electronic patient database and anonymised for further analysis with the purpose of standard‐of‐care service evaluation. According to the National Institute for Health and Care Research in the United Kingdom, retrospective data analysis for service evaluation is absolved from ethics committee approval.

## Consent

No patient identifiable information was used in this publication.

## Conflicts of Interest

The authors declare no conflicts of interest.

## Data Availability

Data are available upon request from the authors, in conformity to national clinical data governance on patient privacy and ethical considerations.

## References

[1] Arnold M. , Morgan E. , Rumgay H et al., Current and Future Burden of Breast Cancer: Global Statistics for 2020 and 2040, Breast. (2022) 66, 15–23, 10.1016/j.breast.2022.08.010. 36084384 PMC9465273

[2] Chen Y. , Shi X. , Tian J. , Yang X. , Wang Y. , and Yang K. , Survival Benefit of Neoadjuvant Chemotherapy for Resectable Breast Cancer: a meta-analysis, Medicine (Baltimore). (2018) 97, no. 20, 10.1097/md.0000000000010634, 2-s2.0-85047337352. PMC5976345 29768327

[3] Spring L. , Fell G. , Arfe A et al., Pathologic Complete Response After Neoadjuvant Chemotherapy and Impact on Breast Cancer Recurrence and Survival: a Comprehensive Meta-analysis, Clinical Cancer Research. (2020) 26, no. 12, 2838–2848, 10.1158/1078-0432.ccr-19-3492. 32046998 PMC7299787

[4] Gianni L. , Pienkowski T. , Im Y. H et al., Efficacy and Safety of Neoadjuvant Pertuzumab and Trastuzumab in Women with Locally Advanced, Inflammatory, or Early HER2-positive Breast Cancer (NeoSphere): A Randomised Multicentre, open-label, Phase 2 Trial, The Lancet Oncology. (2012) 13, no. 1, 25–32, 10.1016/s1470-2045(11)70336-9, 2-s2.0-84855297353. 22153890

[5] Schneeweiss A. , Chia S. , Hickish T. et al., Pertuzumab plus Trastuzumab in Combination with Standard Neoadjuvant anthracycline-containing and Anthracycline-free Chemotherapy Regimens in Patients with HER2-positive Early Breast Cancer: A Randomized Phase II Cardiac Safety Study (TRYPHAENA), Annals of Oncology. (2013) 24, no. 9, 2278–2284, 10.1093/annonc/mdt182, 2-s2.0-84883359410. 23704196

[6] Schmid P. , Cortes J. , Dent R. et al., Overall Survival with Pembrolizumab in Early-Stage Triple-Negative Breast Cancer, New England Journal of Medicine. (2024) 391, no. 21, 1981–1991, 10.1056/nejmoa2409932. 39282906

[7] Pusztai L. , Denkert C. , O’Shaughnessy J et al., Event-Free Survival by Residual Cancer Burden with Pembrolizumab in early-stage TNBC: Exploratory Analysis from KEYNOTE-522, Annals of Oncology. (2024) 35, no. 5, 429–436, 10.1016/j.annonc.2024.02.002. 38369015

[8] Zhu T. , Huang Y. H. , Li W. et al., A Non-invasive Artificial Intelligence Model for Identifying Axillary Pathological Complete Response to Neoadjuvant Chemotherapy in Breast Cancer: A Secondary Analysis to Multicenter Clinical Trial, British Journal of Cancer. (2024) 131, no. 4, 692–701, 10.1038/s41416-024-02726-3. 38918556 PMC11333754

[9] Vikas P. , Korde L. A. , Somerfield M. R. , and Hershman D. L. , Use of Immune Checkpoint Inhibitors in the Treatment of High-Risk, Early-Stage Triple-Negative Breast Cancer: ASCO Guideline Rapid Recommendation Update Q and A, JCO Oncology Practice. (2022) 18, no. 9, 649–651, 10.1200/op.22.00284. 35728019

[10] Loibl S. , André F. , Bachelot T. et al., Early Breast Cancer: ESMO Clinical Practice Guideline for Diagnosis, Treatment and follow-up, Annals of Oncology. (2024) 35, no. 2, 159–182, 10.1016/j.annonc.2023.11.016. 38101773

[11] Kitajima K. and Miyoshi Y. , Present and Future Role of FDG-PET/CT Imaging in the Management of Breast Cancer, Japanese Journal of Radiology. (2016) 34, no. 3, 167–180, 10.1007/s11604-015-0516-0, 2-s2.0-84961206750. 26733340

[12] Kd M. , L N. , Ab M et al., CA: A Cancer Journal for Clinicians, 2019, 69, no. 5, 363–385. 10.3322/caac.21565 31184787

[13] Groheux D. , Vaz S. C. , Ulaner G. A. et al., Joint EANM-SNMMI Guidelines on the Role of 2-[18F]FDG PET/CT in No Special Type Breast Cancer: Differences and Agreements with European and American Guidelines, European Journal of Nuclear Medicine and Molecular Imaging. (2024) 51, no. 9, 2701–2705, 10.1007/s00259-024-06694-x. 38693453

[14] Rco R. , Society B. N. M. , and AoRSA C. , Evidence-Based Indications for the Use of PET-CT in the United Kingdom 2022, 2022, Royal College of Radiologists, London (UK).

[15] Metser U. , Mirshahvalad Sa , Dayes I. , Parpia S. , and Levine M. , Detection of Oligometastatic Disease with 18F-FDG PET/CT in Patients with Locally Advanced Breast Cancer at Initial Staging: A Post-hoc Analysis of a Multicenter Randomized Trial (PET ABC), Journal of Nuclear Medicine. (2025) 66, no. 1.

[16] Health N. If and Excellence C. , Early and Locally Advanced Breast Cancer: Diagnosis and Management, 2023, NICE, London.

[17] Del Mastro L. , Venturini M. , Blondeaux E. et al., Fluorouracil and dose-dense Adjuvant Chemotherapy in Patients with early-stage Breast Cancer (GIM2): End-Of-Study Results from a Randomised, Phase 3 Trial, The Lancet Oncology. (2022) 23, no. 12, 1571–1582, 10.1016/s1470-2045(22)00632-5. 36370716

[18] Tinterri C. , Fernandes B. , Zambelli A. et al., The Impact of Different Patterns of Residual Disease on Long-Term Oncological Outcomes in Breast Cancer Patients Treated with Neo-Adjuvant Chemotherapy, Cancers. (2024) 16, no. 2, 10.3390/cancers16020376. PMC10814808 38254865

[19] P S. , J C. , L P. et al., Pembrolizumab for Early Triple-Negative Breast Cancer, New England Journal of Medicine. (2020) 382, no. 9, 810–821. 32101663 10.1056/NEJMoa1910549

[20] Fukuda T. , Horii R. , Gomi N. et al., Accuracy of Magnetic Resonance Imaging for Predicting Pathological Complete Response of Breast Cancer After Neoadjuvant Chemotherapy: Association with Breast Cancer Subtype, SpringerPlus. (2016) 5, no. 1, 10.1186/s40064-016-1800-x, 2-s2.0-84959232050. PMC4766139 27026849

[21] Kim S. Y. , Cho N. , Park I. A. et al., Dynamic contrast-enhanced Breast MRI for Evaluating Residual Tumor Size After Neoadjuvant Chemotherapy, Radiology. (2018) 289, no. 2, 327–334, 10.1148/radiol.2018172868, 2-s2.0-85055071798. 30152744

[22] McGuire K. P. , Toro-Burguete J. , Dang H. et al., MRI Staging After Neoadjuvant Chemotherapy for Breast Cancer: Does Tumor Biology Affect Accuracy?, Annals of Surgical Oncology. (2011) 18, no. 11, 3149–3154, 10.1245/s10434-011-1912-z, 2-s2.0-83055170003. 21947592

